# Supplementary material for: Dominant resistance and negative epistasis can limit the co-selection of de novo resistance mutations and antibiotic resistance genes
Source: Nat Commun. 2020 Mar 5;11:1199. doi: 10.1038/s41467-020-15080-8 (PMC7057998; doi:10.1038/s41467-020-15080-8)
Supplement: Supplementary file 1 — Supplementary Information [file 41467_2020_15080_MOESM1_ESM.pdf]

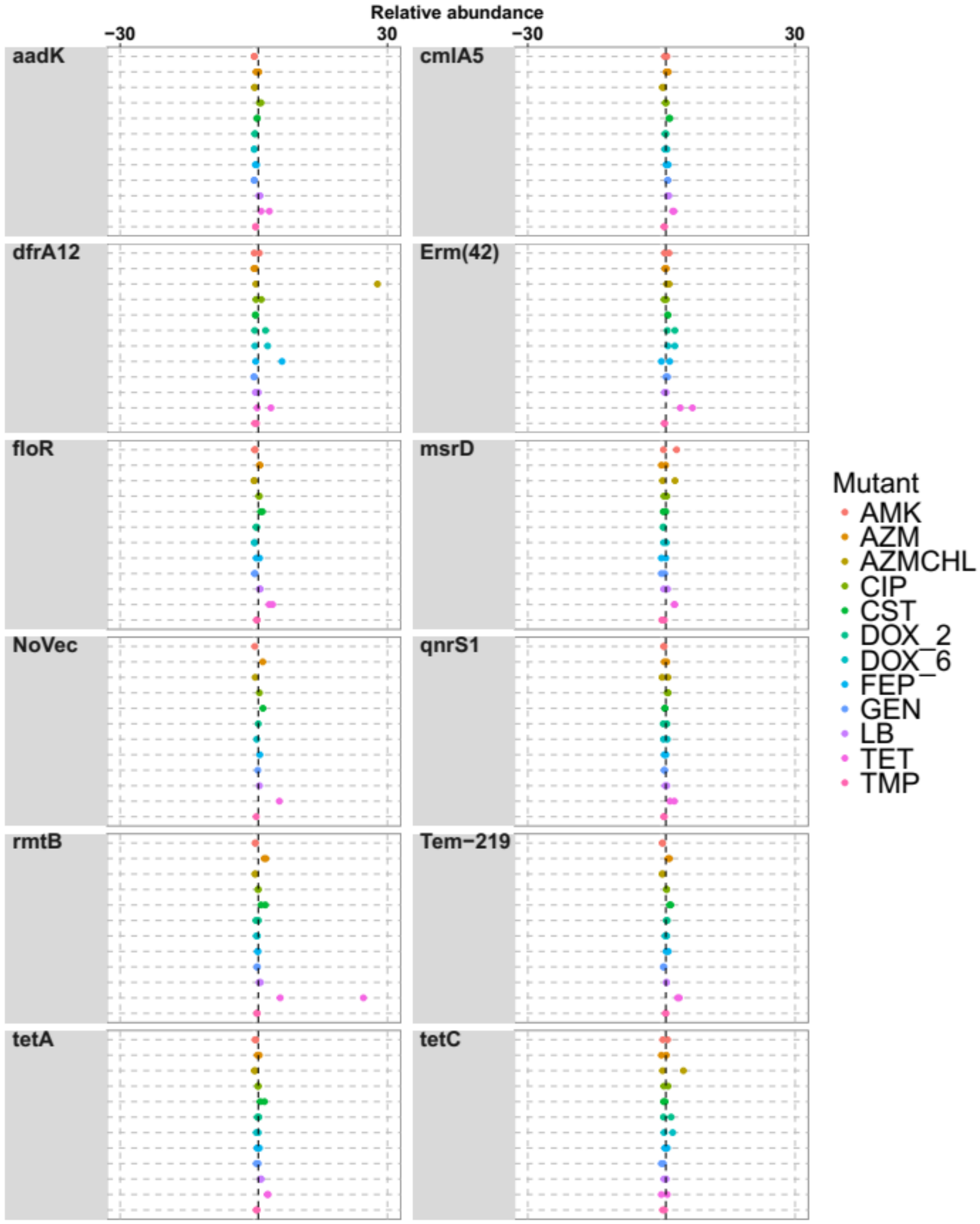

Supplementary Figure 1

ARG-mutant combination fitness without selection. Relative abundances of ARG-mutant combinations after 24h of growth without selection. Normalized to the fitness of mutants carrying the empty vector. There were no significant fitness differences ($p > 0.05$, ANOVA) between mutants carrying the selected ARGs, or those not carrying the vector (NoVec), and those carrying the empty vector backbone.

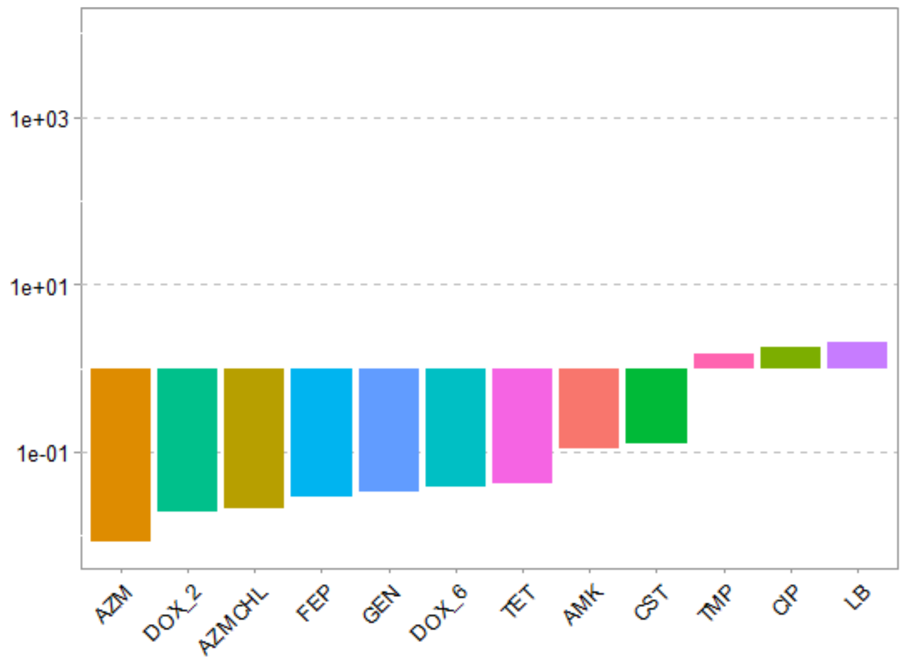

Supplementary Figure 2

Unselected mutant fitness. The relative fitness of each mutant without ARGs after 24 h of growth in the absence of selection. The *y*-axis shows the relative abundance of each mutant.

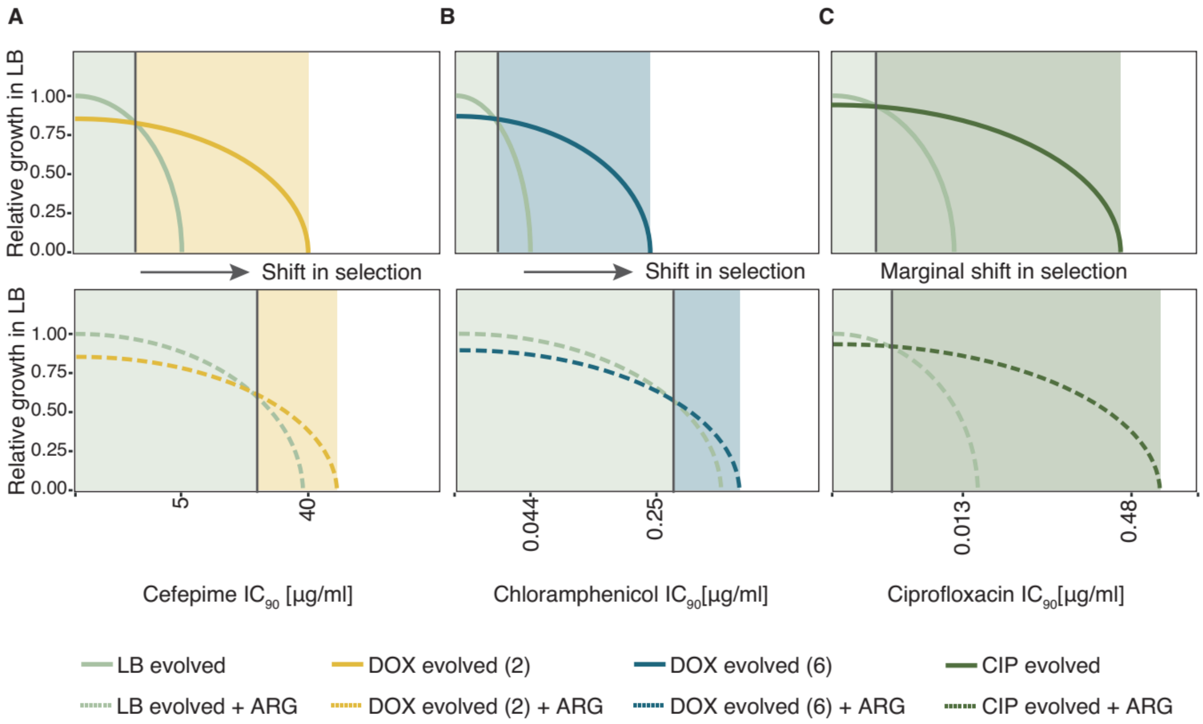

Supplementary Figure 3

Schematic depicting the influence of fitness and resistance level on the preferential selection of ARGs or mutations. (**A**) and (**B**) The WT strain is selected over cefepime/chloramphenicol-resistant mutants at low drug concentrations, where the WT has a fitness advantage. However, the mutant is selected at high concentrations, where its resistance provides an advantage and concentration-dependent increase in fitness. The addition of a resistance gene shifts the resistance level of both the WT and the mutant in an additive manner, whereby the fitness advantage of the WT causes selection of the WT over a wider concentration range. The mutant is selected at only very high concentrations. (**C**) The fitness advantage of the WT without antibiotic exposure over the ciprofloxacin-adapted mutant is smaller than that over most other mutants. In addition, the mutant is highly resistant, while the resistance gene adds only marginal resistance to both the mutant and the WT. While an additive interaction between the resistance gene and the mutant can be detected, only a marginal shift in mutant selection is observed.

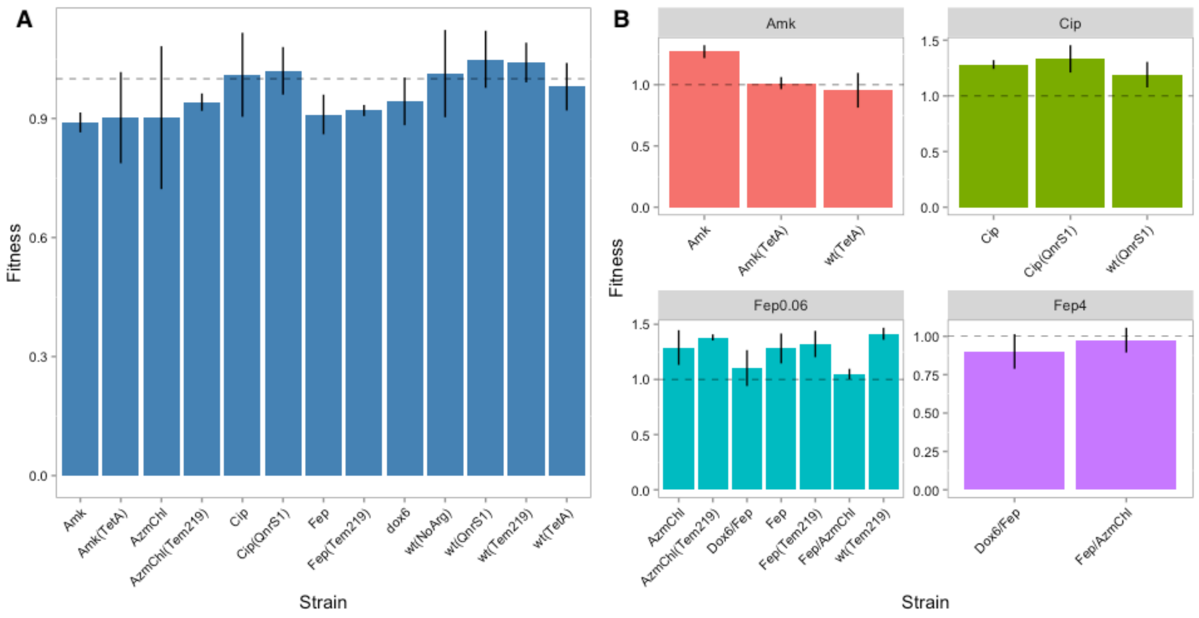

Supplementary Figure 4

Fitness measures resulting from pairwise competition experiments. Each strain was competed against the WT *E. coli* MG1655 for 24h in either LB (**A**) or LB + antibiotics (**B**) and fitness is shown relative to this strain. Additionally, direct competitions of mutants *dox6* against *fep (Dox6/Fep)* and *fep* against *azmchl (Fep/AzmChl)* were performed for the cefepime experiments. The fitness is expressed as the fraction is written. For cefepime, two concentrations of 0.06 µg/ml (turquoise) and 4 µg/ml (purple) were used. For ciprofloxacin and amikacin, the same concentrations as used for the pooled competitions displayed in fig. 2 (0.0075 µg/ml and 16 µg/ml) were used. Error-bars show the standard deviation of three biological replicates. The stippled horizontal line represents the point where the competing strains are equally fit.

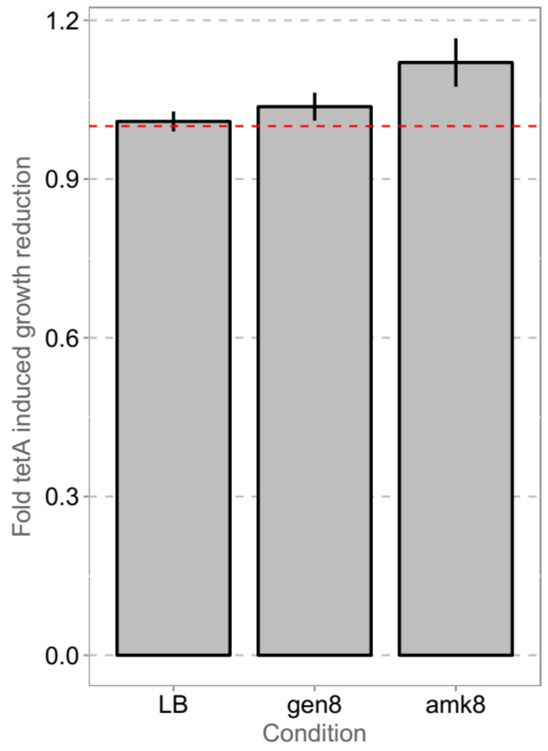

Supplementary Figure 5

Sensitivity of the tetA carrying Amk4 mutant towards sub-MICs of gentamicin and amikacin (8 µg/ml), shown as the impact on growth. The observed sensitivity is only significant for amikacin (Wilcoxon rank sum test, $P < 0.05$). The red dotted line highlights equal fitness (no effect of tetA) and error bars show standard error. While the difference in sensitivity towards different aminoglycosides is not known, we can speculate that the decreased lipid solubility and membrane interaction of streptomycin and amikacin, compared to gentamicin, might play a role, e.g., by increasing dependency on transport mechanisms for uptake of streptomycin compared to the other antibiotics[1].

Supplementary Table 1. Drug-adapted mutant lineages.

Strain	Adapted to	Increase in MIC relative to WT	Mutated genes	Position in protein	Mechanism	PMID
CST	Colistin	45	*basR*	Gly53Glu	two-component system, sensor, kinase, results in positively charged LPS	2862922 9
FEP	Cefepime	22	*acrR, ompC, ompF*	INDEL, Gln171*, INDEL	membrane porin	1910034 6
GEN	Gentamycin	3	*nuoG, fusA*	Ala302fs, Phe593 Leu	proton pump, elongation factor	7515367 2416940 3
AMK	Amikacin	24	*nuoH, lrhA, crr, fusA, rffG, cpxR*	Trp187*, Ala32_Ala34 del, Met1?, Pro610Gln, Gly11Trp, Met53Thr	Membrane potential, stress and elongation factor	7515367 2416940 3
AZM	Azithromycin	9	*acrR, rrlA*	Thr5Ala, INDEL	transcription al regulator for acrAB genes	1218326 2
CIP	Ciprofloxacin	55	*gyrA, parE*	Ser83 Leu, INDEL	DNA gyrase subunit	1535255 1
TMP	Trimethopri m	NA	*folA, rpsC*	Trp30Gly, duplicaion	Folate metabolism	1745144 0
TET	Tetracycline	43	*ybaO, marR, lrhA*	Met107Arg, Leu114*, Gln47Glu	Regulation and efflux	2452377 3

DOX_2	Doxycycline	14	*acrR, marR, rob, trkA*	INDEL, INDEL, Thr184 Lys, INDEL	Transcription factor, stress tolerance	25391482
DOX_6	Doxycycline	46	*lon, arcR, marR, rpsJ*	Pro480 Leu, Phe52 Val, Gly104Asp, Val57 Leu	Protease, regulators of efflux, translation	26989065
AZMCHL	Azithromycin and chloramphenicol	9	*acrR, marR, ompR, rpoB, yrdE, zntR, rplR, rplP, rpsC, yhhY, ryhB*	Leu158 Val, Leu46His, Arg110fs, Arg451Ser, duplication, duplication, duplication, duplication, duplication, duplication, duplication	transcriptional regulator of efflux, porin	29764951
LB	No antibiotics	0	-		-	

Supplementary Table 2. Antibiotic resistance genes.

**Gene**	**Resistance**	**Mechanism**
Tem-219	Beta-lactams	Drug inactivation
Erm(42)	Macrolides, clindamycin	Ribosomal modification
aadk	Aminoglycosides (Str)	Drug inactivation
rmtB	Aminoglycosides (Gen, Str, Amk)	Ribosomal modification
tetA	Tetracycline	Efflux
floR	Chloramphenicol	Efflux
cmlA5	Chloramphenicol	Efflux
qnrS1	Fluoroquinolones	Gyrase protection
drfA12	Trimethoprim	Target replacement
tetC	Tetracycline	Efflux
msrD	Macrolides	Ribosomal protection

Supplementary Table 3. MIC values of mutants derived from adaptations to the drugs used.

Strain	MIC AMK [µg/ml]	MIC AZM [µg/ml]	MIC FEP [µg/ml]	MIC CHL [µg/ml]	MIC CIP [µg/ml]	MIC CST [µg/ml]	MIC TMP [µg/ml]	MIC TET [µg/ml]
**CST**	7.45	6.78	0.015	19.98	0.011	12.74	0.61	0.28
**FEP**	2.08	20.22	0.53	10.98	0.01	0.2	0.35	3.64
**GEN**	8.3	2.3	0.0065	0.89	0.003	0.04	0.07	0.35
**AMK**	72.79	3.94	0.03	1.35	0.025	0.33	0.14	0.52
**AZM**	1.16	39.91	0.042	10.5	0.007	0.15	0.52	1.6
**CIP**	2.91	2.59	0.015	1.86	0.56	0.16	0.12	1.14
**TMP**	3.91	5.50	0.018	2.6	0.007	0.37	NA	0.47
**TET**	4.78	15.76	0.15	26.07	0.04	0.33	0.84	28.61
**DOX_2**	0.42	10.46	0.13	40.1	0.09	0.28	1.01	6.77
**DOX_6**	3.54	24.14	0.25	44.45	0.06	0.39	0.69	22.26
**AZMCHL**	NA	14.19	NA	5.55	NA	NA	NA	NA
**LB (WT)**	6.78	2.68	0.014	3.11	0.007	0.35	0.13	0.6

Supplementary Table 4.

Epistasis values of individual competitions. Epistasis (calculated as: "Fitness of mutant with ARG – fitness of ARG in WT * fitness of mutant") and propagated errors were calculated according to Silva et al. 2011[2]. *P-values* were obtained using a one-sample two-sided *t*-test.

Strain	Epistasis	p-value	Condition
Amk(tetA)	0.030	0.636	LB
Cip(qnrS1)	-0.039	0.341	LB
AzmChl(Tem219)	0.002	0.506	LB
Fep(Tem219)	-0.027	0.284	LB
Amk(TetA)	-0.304	*0.044*	Amk
Cip(QnrS1)	-1.140	*0.005*	Cip
AzmChl(Tem219)	-0.442	*0.049*	Fep (0.06 ug/ml))
Fep(Tem219)	-0.490	*0.029*	Fep (0.06 ug/ml)

Supplementary Table 5. Mutated sequences and MAGE oligos.

The nucleotide reverted by the MAGE procedure is underlined. For *IrhA*, a 9 bp deletion had occurred. The sequences of *tetA*-carrying colonies that had lost their sensitivity to streptomycin had all reverted to the WT sequences of the *nuoH* gene (nucleotide 561 changed from T to C, resulting in a stop codon), but none of the other alleles had changed.

**Sequences of WT alleles used for MAGE**	**gene**	**position**	**AA change**
TGATCTTCTGGACGACAGCATTGATTTACTTTTGCTTGACGTAATGATGCCGAAG AAAAATGGTATCGACACATTAAAAGCACTTCGCCA	*cpxR*	4105525	T53->M
CACCGCGTCGCTCGTTTCGTTGATGATATAACGCACCAGCGCCGAGCCAATAAAC CCGGCACCACCTGTTATCAGAATTTTTCTCATCAG	*rffg*	3972564	W11->G
ACGTCACCGGTGTTCTCTTCCGGAGTTTCTACTTCAACCTTCATGATCGGCTCAAG CAGAACTGGTTTCGCTTTCTTAAAGCCTTCTTTA	*fusA*	3471698	P624->Q
TGCTAATCCACGAGATGCGGCCCAATTTACTGCTTAGGAGAAGATCATGGGTTTG TTCGATAAACTGAAATCTCTGGTTTCCGACGACAAGAAG	*crr*	2535848	M1I
GCATTTGCTGACTTACGGCGGACTGAGTACGACACACAGCGGCAGCTGCGGCAG CAAAAGTGTTCAGATCGGCAACAGCAACAAATGTTCTC	*IrhA*	2406554	ΔTCTTCAATG
CGATGGCAAAGGTAATAAAACCAAAGAATTGCGGGATAACGTTCCACACATGCG CCTGGCTGTTGACGATGTCGGTCATGTTGAATGAACCGG	*nuoH*	2396895	W186->*

Supplementary Table 6. Antibiotics and concentrations used.

Antibiotic	Abbreviation	Concentration range [µg/ml]	Concentrations chosen for sequencing [µg/ml]	Clinical breakpoint [µg/ml] according to EUCAST
Amikacin	Amk	0.5 to 512	16; 256	16
Azithromycin	Azm	0.5 to 512	8; 512	NA
Cefepime	Fep	0.0075 to 7.68	0.06; 3.84	4
Chloramphenicol	Chl	1 to 1024	16; 256	8
Ciprofloxacin	Cip	0.0019 to 1.92	0.0075; 0.48	0.5
Colistin	Col	0.0625 to 64	1; 4	2
Tetracycline	Tet	0.125 to 128	2; 128	NA
Trimethoprim	Tmp	0.0625 to 64	8; 64	4

References:

1. Brasseur, R., Laurent, G., Ruysschaert, J. M. & Tulkens, P. Interactions of aminoglycoside antibiotics with negatively charged lipid layers. Biochemical and conformational studies. *Biochem. Pharmacol.* **33,** 629–637 (1984).

2. Silva, R. F. *et al.* Pervasive sign epistasis between conjugative plasmids and drug-resistance chromosomal mutations. *PLoS Genet.* **7,** e1002181 (2011).
